# Associations Between Environmental and Sociodemographic Data and Hepatitis‐A Transmission in Pará State (Brazil)

**DOI:** 10.1029/2020GH000327

**Published:** 2021-05-01

**Authors:** Philipe Riskalla Leal, Ricardo José de Paula Souza e Guimarães, Milton Kampel

**Affiliations:** ^1^ National Institute for Space Research (INPE, Instituto Nacional de Pesquisas Espaciais) São Paulo Brazil; ^2^ Evandro Chagas Institute Belém State of Pará Brazil

**Keywords:** geoprocessing, hepatitis‐A transmission modelling, remote sensing, time‐space epidemiology analyses

## Abstract

Hepatitis‐A is a waterborne infectious disease transmitted by the eponymous hepatitis‐A virus (HAV). Due to the disease's sociodemographic and environmental characteristics, this study applied public census and remote sensing data to assess risk factors for hepatitis‐A transmission. Municipality‐level data were obtained for the state of Pará, Brazil. Generalized linear and nonlinear models were evaluated as alternative predictors for hepatitis‐A transmission in Pará. The Histogram Gradient Boost (HGB) regression model was deemed the best choice (RMSE= 2.36, and higher $R^{2}$ = 0.95) among the tested models. Partial dependence analysis and permutation feature importance analysis were used to investigate the partial dependence and the relative importance values of the independent variables in the disease transmission prediction model. Results indicated a complex relationship between the disease transmission and the sociodemographic and environmental characteristics of the study area. Population size, lack of sanitation, urban clustering, year of notification, insufficient public vaccination programs, household proximity to open‐air dumpsites and storm‐drains, and lack of access to healthcare facilities and hospitals were sociodemographic parameters related to HAV transmission. Turbidity and precipitation were the environmental parameters closest related to disease transmission. Based on HGB model, a hepatitis‐A risk map was built for Pará state. The obtained risk map can be thought of as an auxiliary tool for public health strategies. This study reinforces the need to incorporate remote sensing data in epidemiological modelling and surveillance plans for the development of early prevention strategies for hepatitis‐A.

## Key Points

- Hepatitis‐A is a waterborne infectious disease responsible for ∼70,000 deaths per year around the world
- In this work, sociodemographic and environmental factors were related to hepatitis‐A transmission by applying census and remote sensing data
- This research stresses the need to incorporate remote sensing data to epidemiological modelling for prevention and surveillance plans

## 1 Introduction

Risk assessment and vulnerability analyses are common practices in epidemiology (Avanzi et al., 2018; Gullón et al., 2017; WHO, 2014). Evidence from around the world confirms that climate change can affect distribution and occurrence of diseases, a major concern for policy making and healthcare facilities (UN, 2007). The health of human populations is sensitive to shifts in weather patterns and other aspects of climate change (Smith et al., 2015). Weather events and climate change are important drivers of the transmission of waterborne diseases—for instance, cholera, dysentery, and waterborne hepatitis are expected to have higher incidence, or even spread to new areas (Ahern et al., 2005; Davies et al., 2015).

Most of the burden of climate change will be borne by developing countries, where the incidence of viral hepatitis and other communicable diseases has traditionally been high, and where healthcare systems still lack proper coverage for health‐related products and services (Carballo et al., 2013). Previous reports have indicated that the main causes of waterborne diseases are related to contamination of water supply systems, usually through increased run‐off from surrounding areas or by inundation (Cann et al., 2013). Nevertheless, other factors, for example, climate variability, also influence waterborne disease transmission (WHO, 2009).

Hepatitis‐A is an infectious disease transmitted by the eponymous hepatitis‐A virus (HAV) and accounts for ∼70,000 deaths per year around the world (WHO, 2016). Hepatitis‐A may cause debilitating symptoms and lead to acute liver failure, which is associated with high mortality (WHO, 2019). HAV transmission occurs in different ways, though the fecal‐oral route is the most common worldwide (Fiore et al., 2006). Fecal‐oral transmission occurs when a susceptible person has direct contact with an infectious person or ingests contaminated food or water (WHO, 2011). The latter transmission route is intimately dependent on sanitary, social, cultural and environmental conditions (Clemens et al., 2000; Fiore et al., 2006; Jacobsen & Koopman, 2005; MS, 2005; Nunes et al., 2016; Pereira & Gonçalves, 2003).

Previous studies have indicated that hepatitis‐A transmission may be related to extreme precipitation and flooding events (Gullón et al., 2017; Marcheggiani et al., 2010). In Brazil, extreme precipitation events have been positively related to HAV outbreaks (Santos et al., 2019). In Spain, intense rainfall has also been associated with greater incidence of hepatitis‐A (Gullón et al., 2017). From a climate change perspective, one may expect more intense and more frequent precipitation events in the future (Camuffo et al., 2018; UN, 2007). This fact and how it bears upon epidemiological outbreaks pose a great challenge for policy making, public health agencies and management planning (Marcheggiani et al., 2010).

Prior to the year 2002, Brazil was considered highly endemic for HAV infection (Souto et al., 2019). It was characterized by mostly affecting children, adolescents and young adults heterogeneously spread throughout the country (Clemens et al., 2000; MS, 2002, 2018). The incidence was steady in 6,000 cases per year until 2014. Between 2014 and 2016, there has been an 85.5% cumulative drop, independent of gender and geographical regions, after the introduction of single‐dose HAV vaccine program in the National Vaccination Calendar of the Unified Health System (SUS), Brazil's public health system (MS, 2014; Souto et al., 2019). Specifically, in the northern region, the HAV‐related mortality rate has been increasing since 2013. Between 2012 and 2016, the HAV mortality coefficient doubled, reaching 35 cases per million inhabitants (MS, 2018).

Given the importance of effective prevention and control of hepatitis‐A in Brazil and similar places, assessment of the main factors associated with disease transmission is paramount. In order to determine where disease‐favoring conditions are present in the environment, remote sensing can be of great importance to assess disease‐related environment factors (Patel, 2020). This assessment can provide meaningful insights for controlling disease transmission.

In light of the topics above, this study assessed how hepatitis‐A transmission relates to environmental data detectable by remote sensing and to sociodemographic data derived from the national census and from vaccination programs of the state of Pará (in the Amazon region), Brazil. Various models were tested to best identify and characterize the main variables associated with the hepatitis‐A transmission. A municipality grid was applied to perform the spatial aggregation among the data sets.

## 2 Material and Methods

### 2.1 Study Area

**Figure 1:**
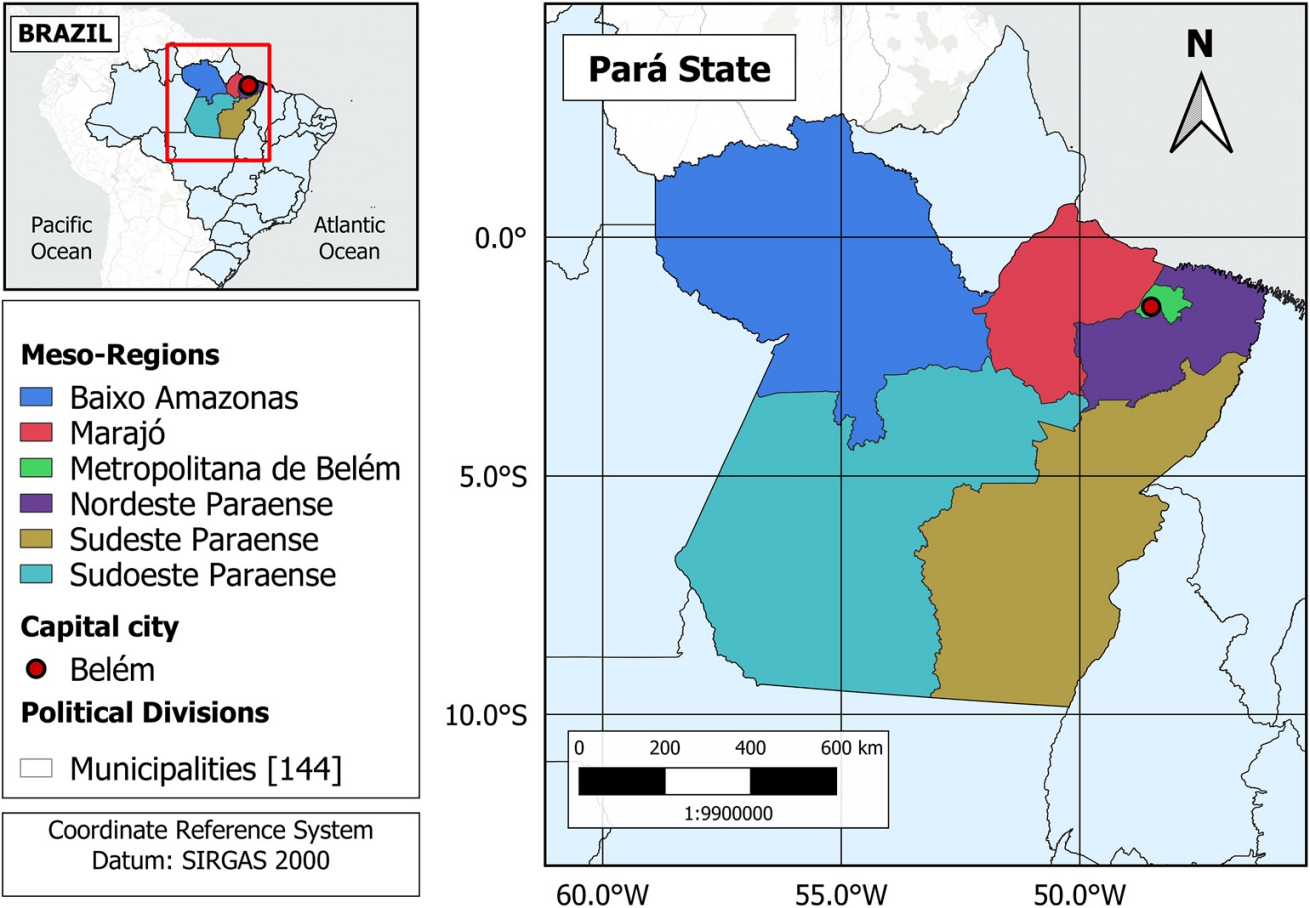
Study area: state of Pará (Brazil). Geographical definitions by the Brazilian Institute of Geography and Statistics (IBGE) (IBGE, 2019).

Epidemiological, sociodemographic and remote sensing data were obtained for the northern state of Pará, Brazil. In this region, floods are gradual and natural to the ecosystem dynamics (IBGE, 2019). The state of Pará comprises 144 municipalities and six mesoregions (Figure 1). The geographical limits of the municipalities were obtained from the Brazilian Institute of Geography and Statistics (IBGE) (IBGE, 2019) and their grid was applied to spatially integrate the different data sets of this study. The municipality was the political unit of choice, being the smallest political‐administrative unit of the Brazilian federative republic (Ramalho, 2020).

### 2.2 Epidemiological Data

Information on hepatitis‐A cases was obtained from the Notifiable Diseases Information System (SINAN) of Brazil's Ministry of Health (MS, 2007). Data included individual names and addresses, all of which were omitted to ensure and preserve confidentiality, and comprised Pará's residents confirmed to be infected with the hepatitis‐A virus between January 2008 and December 2017. The data was aggregated by municipality and month. The epidemiological data set was geocoded by municipality and consists of 5,500 reported positive new cases (RPC), representing 4.26% of all RPCs in Brazil.

### 2.3 Sociodemographic Data

Annual data on the coverage of the anti‐HAV vaccination program and on the number of live births in each municipality were obtained from the SUS's Information Technology Department platform (DATASUS) (MS, 2019), encompassing annual vaccination rates per municipality for the 2014–2017 period. Population coverage of anti‐HAV vaccination is the ratio between vaccinated individuals (infants and children under 2) and the total population of a given municipality.

A total of eight variables were obtained from the IBGE, (2011) census data (IBGE, 2011): households with/without sanitation; households near storm drains; households near open‐air sewage discharge; households near open‐air dumpsites; households with running water; households with water‐wheel; and households with a self‐supplied water. These census data indicate the number of households in each condition. Therefore, each variable was transformed into relative percentages by dividing the number of households by the total number of households in each municipality. The annual population estimate per municipality was also obtained from the IBGE (IBGE, 2017). The demographic data was applied to evaluate the incidence of the disease in each municipality. A temporal dependence was also incorporated to the model by adding the covariate “year”. The variable reflects the year of notification of each reported case of hepatitis‐A in the epidemiological data of the SINAN.

All geographical and political boundaries and shapes (municipalities and mesoregions) were obtained from the IBGE (IBGE, 2019). The municipalities' centroid coordinates (longitude and latitude) were taken as covariates during the modelling, enabling the integration of spatial dependence into the models. The municipalities' centroid coordinates were previously reprojected for the SIRGAS 2000 polyconic projection.

### 2.4 Environmental Data

The Google Earth Engine (GEE) platform allows easy access to several global remote sensing data sets thanks to the computational processing power of Google servers (Gorelick et al., 2017). The platform was used to retrieve environmental variables detectable by remote sensing pertaining to hepatitis‐A modelling. For the present study, eight variables were selected: surface daytime temperature (SDT), surface nighttime temperature (SNT), turbidity, total suspended matter (TSM), enhanced vegetation index (EVI), normalized difference index (NDVI), precipitation, and hydrological mobility index (HMI) (see Table 1). All remote sensing variables were aggregated monthly over the study period (2008–2017).

**Table 1:** General Characteristics of the Remote Sensing Variables Used in This Study (Data Access: Google Engine Platform)

Data	Source	Sensor	Spatial resolution	Spatial aggregation	Temporal resolution
Daytime and nighttime surface temperature	NASA^a^/USGS^b^	MODIS^c^	1 × 1 km	Average per municipality	8 days
Surface spectral reflectance^d^	NASA^a^/USGS^b^	Landsat series	30 × 30 m	Average per municipality	16 days
EVI/ NDVI	NASA^a^/USGS^b^	MODIS^c^	250 × 250 m	Average per municipality	16 days
Altimetry	SRTM^e^	Radar	30 × 30 m		
Precipitation	Climate Hazards Group	Multi‐plataform^f^	4 × 4 km	Average per municipality	Daily

^a^ NASA: National Aeronautics and Space Administration.

^b^ USGS: United States Geological Survey.

^c^ MODIS: Moderate Resolution Imaging Spectroradiometer.

^d^ Landsat surface spectral reflectance atmospherically corrected by the LASRC algorithm (U.S. GEOLOGICAL SURVEY, 2019).^33^

^e^ SRTM: ‐ Shuttle Radar Topographic Mission (de saint‐exupéry et al., 2007).^34^

^f^ Precipitation from the Climate Hazards Group Infrared Precipitation with Stations (CHIRPS) data set. (Funk et al., 2015) The data set comprises different platforms, orbiting sensors and in situ meteorological station data.

Data on surface daytime and nighttime temperatures (SDT and SNT, respectively) were derived from the Moderate Resolution Imaging Spectroradiometer (MODIS), product MOD11A2, with 1 km^2^ spatial resolution (Wan et al., 2015). *SDT* and *SNT* are important factors that induce human behavior, as fluctuations in their values indirectly influence human activities such as bathing, hydration and water recreation (Parsons, 2003). Thus, one should expect oscillations in daily temperatures to influence HAV transmission.

The Landsat surface reflectance data set was used to estimate the TSM and the turbidity of the waterbodies of each municipality in Pará. These water quality parameters include water transparency (Alcântara; Curtarelli; Stech, 2016b; Ody et al., 2016; Rodrigues et al., 2017), transmittance (Lee et al., 2015), and, consequently, the amount of solar irradiance available in the system. Since solar irradiance directly influences virus survival in aquatic systems through photodegradation (Bales et al., 1993; Hu et al., 2015; Mavignier & Frischkorn, 1992; Sattar et al., 2000), TSM and turbidity are expected to be indirectly related to HAV survival, and therefore, to viral transmissivity.

Turbidity was estimated using a semi‐empirical algorithm previously validated for both estuarine and coastal waters (Dogliotti et al., 2014). The algorithm relates turbidity to remote sensing reflectance at wavelength (λ), with ${\rho_{w}}_{\left(\lambda \right)}$. ${\rho_{w}}_{\left(\lambda \right)}$ is defined as the ratio of water‐leaving radiance ($L_{w_{\left(\lambda \right)}}$) and the above‐water downwelling irradiance ($E_{0{+}_{\left(\lambda \right)}}$). The resulting turbidity is expressed in Formazin Nephelometric Units (FNU). The algorithm was validated for independent environments, with stable performance and relative mean error below 13.7%. The algorithm is described in Equations 1, 2 and 3. $A_{\left(\lambda \right)}$ and $C_{\left(\lambda \right)}$ are spectral conditional constants that follow the conditional rules from Equation 3. w is a linear mixture factor for cases in which ${\rho_{w}}_{\left(\lambda \right)}$ is between 0.05 and 0.07 (sr^−1^).

(1) $$T\left({\rho_{w}}_{\left(\lambda \right)}\right)=\;\frac{A_{\left(\lambda \right)}*\;{\rho_{w}}_{\left(\lambda \right)}}{\frac{\left(1-\;{\rho_{w}}_{\left(\lambda \right)}\right)}{C_{\left(\lambda \right)}}}$$

(2) $$w=\left[\frac{{\rho_{w}}_{\left(\lambda =645\right)}-0.05}{0.02}\right]$$

(3) $$T=\left\{\begin{array}{l} T\left({\rho_{w}}_{\left(\lambda =645\right)}\right),\;{\rho_{w}}_{\left(\lambda =645\right)}<0.05\;∴\;A_{\left(\lambda \right)}=228.1,\;C_{\left(\lambda \right)}=0.1641\\ T\left({\rho_{w}}_{\left(\lambda =859\right)}\right),\;{\rho_{w}}_{\left(\lambda =859\right)}\geq 0.07\;∴\;A_{\left(\lambda \right)}=3078.9,\;C_{\left(\lambda \right)}=0.2112\\ \left(1\;-w\right)\cdot \;T\left({\rho_{w}}_{\left(\lambda =645\right)}\right)\;+w\;\cdot \;T\left({\rho_{w}}_{\left(\lambda =859\right)}\right)\;,\;0.05\leq \;{\rho_{w}}_{\left(\lambda =645\right)}<0.07\;∴\; \end{array}\right.$$

TSM was estimated using a generalized algorithm validated for continental waters (Alcântara et al., 2016a). The algorithm has been previously validated with performance values for root mean square error (RMSE) equal to 24.62 (Alcântara et al., 2016a). The algorithm defines TSM as a second degree polynomial function of the ratio of two remote sensing reflectances. For this study, the algorithm was applied to the MODIS data set, given its higher temporal resolution (daily) vis‐à‐vis Landsat's (∼16 days). Therefore, the spectral bands were corrected to the nearest available band from MODIS (see Equation 4 and Equation 5).

(4) $$TSM=\;0.03*\;index^{2}-\;0.08*\;index\;+\;0.9$$

(5) $$index=\;{\rho_{w}}_{\left(\lambda =555\right)}/{\rho_{w}}_{\left(\lambda =469\right)}$$

Since water body dynamics is mainly influenced by climatic and inter‐annual variability (i.e., tides, rain cycles, temperature oscillations) (Simons & Sentürk, 1976), as well as by land use and land coverage changes that directly impact transport of sediments, deposition of materials and biochemistry fluxes (Simons & Sentürk, 1976), EVI and NDVI were also integrated into the model. Both indexes can be related to surface vegetation coverage (da Silva et al., 2019) and both were derived from MODIS product MOD13Q1, with 1 × 1 km spatial resolution (Justice et al., 1998).

Data from the Climate Hazards Group Infrared Precipitation with Station (CHIRPS) (Funk et al., 2014) were applied to assess monthly accumulated precipitation in the municipalities of Pará. CHIRPS data have a spatial resolution of ∼5.6 × 5.6 km^2^ and encompass nearly 30 years of quasi‐global rainfall data (50°S–50°N). CHIRPS provides gauge‐precipitation satellite estimates with low latency, high resolution, low bias, and long record period (Funk et al., 2015).

The digital elevation data set from the Shuttle Radar Topography Mission (SRTM) (SRTM, 2015) and the CHIRPS precipitation data set were used to estimate the Hydrological Mobility Index (HMI). Both data sets were spatially resampled to the same spatial resolution of the CHIRPS data set (which has coarser spatial resolution). The index describes the hydrological flushing potential of a given surface (Fonseca et al., 2007) and, thus, can be associated with pathogen dispersal in the environment, serving both as a flusher and a retainer of the virus, influencing disease transmission (Barbosa et al., 2017; Fonseca et al., 2007).

Another five environmental variables were also later derived from the CHIRPS data set to be incorporated in the hepatitis‐A modelling: PPF1.0%, PPF5.0%, PPF90.0%, PPF99.0% and PPF99.9%, where PPF stands for point‐probability function. Each PPF represents the cumulative number of monthly precipitation occurrences given an intensity threshold that might be expected from the PPF of a predefined family of probability distribution functions (PDF). The PPF approach was applied to evaluate the potential relationship between disease transmission and extreme precipitation events (Diaz & Murnane, 2008; Gullón et al., 2017; Marcheggiani et al., 2010). Since there is still much to be considered with respect to extreme precipitation events, this statistical approach was based on prior similar epidemiological studies (Curriero et al., 2001; Gullón et al., 2017). In brief, the algorithm for the derivation of these secondary precipitation variables can be described in three steps, as follows:

First, the precipitation time‐series is linearly decomposed into three time components: the trend ($T_{\left(t\right)}$), the seasonal ($S_{\left(t\right)}$) and the residue ($R_{\left(t\right)}$). This approach assumes that the trend changes linearly over time, implying a linear additive structure (Equation 6). In addition, the decomposition assumes that seasonality presents constant frequency (width of cycles) and amplitude (height of cycles) over time.

(6) $$Y_{\left(t\right)}=\;T_{\left(t\right)}+S_{\left(t\right)}+R_{\left(t\right)}$$

Second, a Pearson Type III probability distribution family is fit into $R_{\left(t\right)}$ by means of a Maximum Likelihood Estimation (MLE) (Virtanen et al., 2020). This *PDF* family is defined in terms of the mean (μ), the standard deviation (σ) and the skewness (*skew*) of the distribution (Vogel & McMartin, 1991) (Equation 7). This produces a large number of different distributions, both skewed and symmetrical, and is reduced to a standard frequency function when skewness is zero. This type of distribution is largely used by the U.S. Army Corps of Engineers in flood frequency analysis, by the National Oceanic and Atmospheric Administration in precipitation data analysis, and by the U.S. Navy (Federal Aviation Administration (FAA), 2003).

(7) $$\text{PDF}\left(x|\;skew,\;\sigma ,\;\text{µ}\right)=\frac{\left|\beta \right|}{\Gamma_{\left(a\right)}}*{\left[\beta *\left(x-\;\zeta \right)\right]}^{\left(a-1\right)}*\exp\left[-\beta *\left(x-\;\zeta \right)\right]$$

where,

(8) $$\beta =\;\frac{2}{skew*\sigma }$$

(9) $$a={\left(\sigma *\;\beta \right)}^{2}$$

(10) $$\zeta =\;\text{µ}-\left(\frac{ɑ}{\beta }\right)$$

(11) $$\Gamma_{\left(x\right)}=\;\overset{\infty }{\underset{0}{\int }}t^{\left(x-1\right)}*e^{\left(-t\right)}dt$$

Finally, skew and σ are the skewness and the standard deviation of the time‐series, respectively.

Once the PDF is fitted for$\;R_{\left(t\right)}$, its hyper‐parameters as well as the selected percentiles (1.0%, 5.0%, 90.0%, 99.0%, and 99.9%) are used to retrieve thresholds for later classification of $R_{\left(t\right)}$. The thresholds are then assessed by means of the point probability function ($PPF_{\left(x\right)}$) of the given PDF. PPF is defined as the inverse of a cumulative distribution function (CDF). PPF is also called probability quantile function in statistics literature (Wasserman, 2009), but the PPF nomenclature is used here. The Pearson Type III PPF is defined in Equation 12.

(12) $$PPF_{\left(q|skew,\;\sigma ,\;\text{µ}\right)}=\;CDF_{\left(q|ɑ,\beta ,\zeta \right)}^{-1}=\frac{1}{\Gamma_{\left(a\right)}}*\frac{\left[\int_{0}^{q}t^{\left(a-1\right)}*e^{\left(-t\right)}dt\right]}{\beta }+\;\zeta$$

For the third and final step of the algorithm, the thresholds derived from Equation 12 are then used to classify$\;R_{\left(t\right)}$. The classified $R_{\left(t\right)}$ is then aggregated monthly for each threshold. These parameters are used as proxies for the evaluation of precipitation disaster events, since they can be highly significant for waterborne diseases such as hepatitis‐A (Freitas et al., 2015).

### 2.5 Data Pre‐processing

Prior to analyzing the data, all variables and all hepatitis‐A cases were aggregated per municipality and per month. Remote sensing variables were averaged per month and per municipality. Precipitation data were summed monthly and averaged spatially for each municipality. Elevation and declivity data were averaged spatially for each municipality.

### 2.6 Statistical Analyses

Multivariate regression analyses were used to evaluate the best model for assessing the main factors that impact hepatitis‐A transmission. The evaluated regression models used here were: a) the Generalized Linear Model (GLM); b) the Multilayer Perceptron (MPL) deep‐learning algorithm; c) the Gradient Boost (GB); d) the Decision Tree (DT); e) the Histogram Gradient Boost (HGB). All algorithms are implemented in the Python's Statsmodels package (Pedregosa et al., 2011).

In the GLM model, the Poisson and Negative Binomial (NB) probability distribution families were used. In the Poisson distribution, each $Y_{\left(i\right)}$ is a random variable in which the Poisson distribution has an expected value (${\text{µ}}_{\left(i\right)}$) (Equation 13) that represents the number of observed events in a given ${\text{municipality}}_{\left(i\right)}$.

(13) $$Yi\;∼\;Poisson\;({\text{µ}}_{\left(i\right)})$$

The expected value (${\text{µ}}_{\left(i\right)}$) was assumed to be the linear sum of each relative risk coefficient ($\theta_{\left(i\right)}$) and the respective linear expected value ($E_{\left(i\right)}$) (Equation 14). In this study, the relative risk coefficient represents the relative increase in hepatitis‐A transmission in $municipality_{\left(i\right)}$, while $E_{\left(i\right)}$ is the expected hepatitis‐A transmission in $municipality_{\left(i\right)}$ under the null hypothesis. Under this hypothesis, the transmission risk of the disease is constant over the entire study area. The relative risk can take on real values between zero and +∞. If the relative risk is 1, this would mean that all verified municipalities have the same average risk of infection in the study area; if less than one, it would mean that the municipality's transmission risk is lower. If higher than one, it would mean that the municipality's transmission risks is higher.

(14) $${\text{µ}}_{\left(i\right)\;}=\;E_{\left(i\right)}\;*\theta_{\left(i\right)}$$

Alternative to the Poisson family distribution, the negative binomial (NB) family is also commonly used to model counting processes, the main difference being that it allows for over‐dispersion of the data. Under this assumption, the data follow an expected value $E\left(Y_{\left(i\right)}\right)=\;\mu_{\left(i\right)}$ and variance $V\left(Y_{\left(i\right)}\right)=\mu_{\left(i\right)}+\;{\left(\mu_{\left(i\right)}\right)}^{2}/\kappa$ (Fox, 2008). Unless the parameter κ is large, the variance of Y increases more rapidly than for a Poisson distributed variable. By defining the expected value of $Y_{\left(i\right)}$ as a random variable, it is possible to incorporate additional variability among observed counts. The PDF of a NB variable is described in Equation 15.

(15) $$p_{Y_{\left(y|\mu ,\;\kappa \right)}}=\;\text{exp}\{\left[\text{ylog}\left(\frac{\mu }{\left(\mu +\;\kappa \right)}\right)-\;\kappa *\log\left(\mu +\;\kappa \right)\right]+\;\kappa *\log\left(\kappa \right)+\log\left(\Gamma_{\left(\kappa +y\right)}\right)-\log\left(\Gamma_{\left(\kappa \right)}\right)-\log\left(y!\right)$$

The MPL algorithm is a nonlinear model. It assumes that the relationship between the covariates and the dependent variable can be defined by an association of neurons structured in sequential layers (de Wilde, 2013). The MPL algorithm accepts several types of activation functions (log−loss, identity, tanh, relu, etc.). In this study, the relu activation function (Equation 16) together with stochastic gradient descent adam solver (Kingma & Ba, 2015) were applied to evaluate the weights of the neuron matrix.

(16) f(x)=max(0,x)

Gradient Boost (GB), Decision Tree (DT) and Histogram Gradient Boost (HGB) are machine learning (ML) algorithms that can perform both classification and regression tasks. They are capable of fitting complex data sets in an additive model approach (Boehmke & Greenwell, 2019). These ML algorithms can capture nonlinear relationships between the covariates and the dependent variable in forward stage wise fashion (Petrere & Friedman, 2000) by minimizing the negative gradient of a given loss function (Pedregosa et al., 2011). Machine learning is greatly influenced by its hyper‐parameters setting. Therefore, tuning these hyper‐parameters is an essential step in analysis. For each model, a grid‐search technique (Unpingco, 2016) was applied to retrieve the respective best fitting hyper‐parameters of each model configuration. The RMSE loss function (Equation 17) was applied to fit each model and, respectively, select the best hyper‐parameters.

After fitting the different ML models, each had its coefficient of determination $R^{2}$ (Equation 19) evaluated. Only the models with a strictly positive (above zero) coefficient of determination were selected, discarding those with negative $R^{2}.$ This initial model filtering step was required in order to minimize potential overfitting (therefore bias) in the models' tunings (Boehmke & Greenwell, 2019). After this filtering step, the remaining models were cross‐compared in respect to their RMSE, and the best model was deemed the one with lowest RMSE.

(17) $$RMSE=\;\sqrt{\frac{\sum_{i=1}^{n}\left[{\left({\hat{y}}_{i}-\;y_{i}\right)}^{2}\right]}{n}}$$

(18) $$MSE=\frac{\sum_{i=1}^{n}\left[{\left({\hat{y}}_{i}-\;y_{i}\right)}^{2}\right]}{n}$$

(19) $$R^{2}=\left(1-\;\frac{MSE}{\sum_{i=1}^{n}{\left[\left(y_{i}-\;E(y\right)\right]}^{2}}\right)=1-\;\frac{MSE}{TSS}$$

After selecting the best regression model for the number of cases of hepatitis‐A (the one with the lowest RMSE), a partial dependence analysis (PDA) and the permutation feature importance (PFI) were verified. The PDA can depict the relationship between the dependent and the independent variables of the model (Molnar, 2019). It graphically structures the variables' marginal effects (whether linear, monotonic or more complex) (Petrere & Friedman, 2000). PFI is a model inspection technique especially useful for nonlinear/complex estimators (Pedregosa et al., 2011) and is defined as the decrease in a model score (e.g., RMSE) when a single covariate is randomly shuffled (Pavlov, 2019). A shuffling effort of 99 shuffles was applied for the PFI analysis.

A spatial analysis was applied for evaluation of the best regression model's predictions (and respective residues) in regards to the reported notification cases of hepatitis‐A. These variables were interpolated to a continuous surface covering the study area, and later averaged over time for visual inspection. The kernel density estimate (KDE) interpolation method was applied for generating the respective continuous surfaces. The Seaborn python's Package KDE's algorithm (Waskom; The Seaborn Development Team, 2020) was applied for the interpolations.

## 3 Results

A set of six different techniques was applied to model hepatitis‐A transmission. Of all models tested (Table 2), HGB Regression proved to be the best in terms of RMSE and $R^{2}$ criteria. GB obtained the lowest RMSE of all models, despite its low non‐biased$\;R^{2}$. GLM‐Poisson, MPL, and DT returned negative $R^{2}$ scores, indicating biased estimates. The set of optimized hyper‐parameters derived from the grid‐search analysis can be found in Table 3.

**Table 2:** Relationship of the Best‐Fitted Models With Respective Residual Fitness

Models	RMSE	$R^{2}$	$R^{2}$ adjusted	Fitting time (s)	Log‐likelihood	Deviance	$\chi^{2}$
GLM ‐ Poisson	11.311	−3.399	0.331	0.112	$-7.27*10^{4}$	$1.27*10^{5}$	$2.78*10^{5}$
GLM – NB	168.477	0.010	0.323	1.050	$-2.87*10^{4}$	$2.862*10^{4}$	$6.39*10^{4}$
MPL	0.100	−249.366	N/A	3.288	N/A	N/A	N/A
GB	0.094	0.126	N/A	3.543	N/A	N/A	N/A
DT	0.000	−6.061	N/A	0.145	N/A	N/A	N/A
HGB	2.358	0.953	N/A	2.843	N/A	N/A	N/A

**Table 3:** Best Hyper‐Parameter Settings of the Grid Search Analyses of Each Tested Model

Models	HL	Learning rate	Leaf size	Min samples per leaf	Nearest neighbors	Max depth	N estimators
GLM	N/A	N/A	N/A	N/A	N/A	N/A	N/A
MPL	( 3, 4)	0.001	N/A	N/A	N/A	N/A	N/A
GB	N/A	0.1	N/A	N/A	N/A	17	188
DT	N/A	N/A	N/A	N/A	N/A	N/A	N/A
HGB	N/A	0.05	150	13	N/A	20	N/A

*Note*. HL: hidden layers ‐ (N° of neurons per layer); “N/A” indicates a hyper‐parameter that is not applicable to a given model.

**Figure 2:**
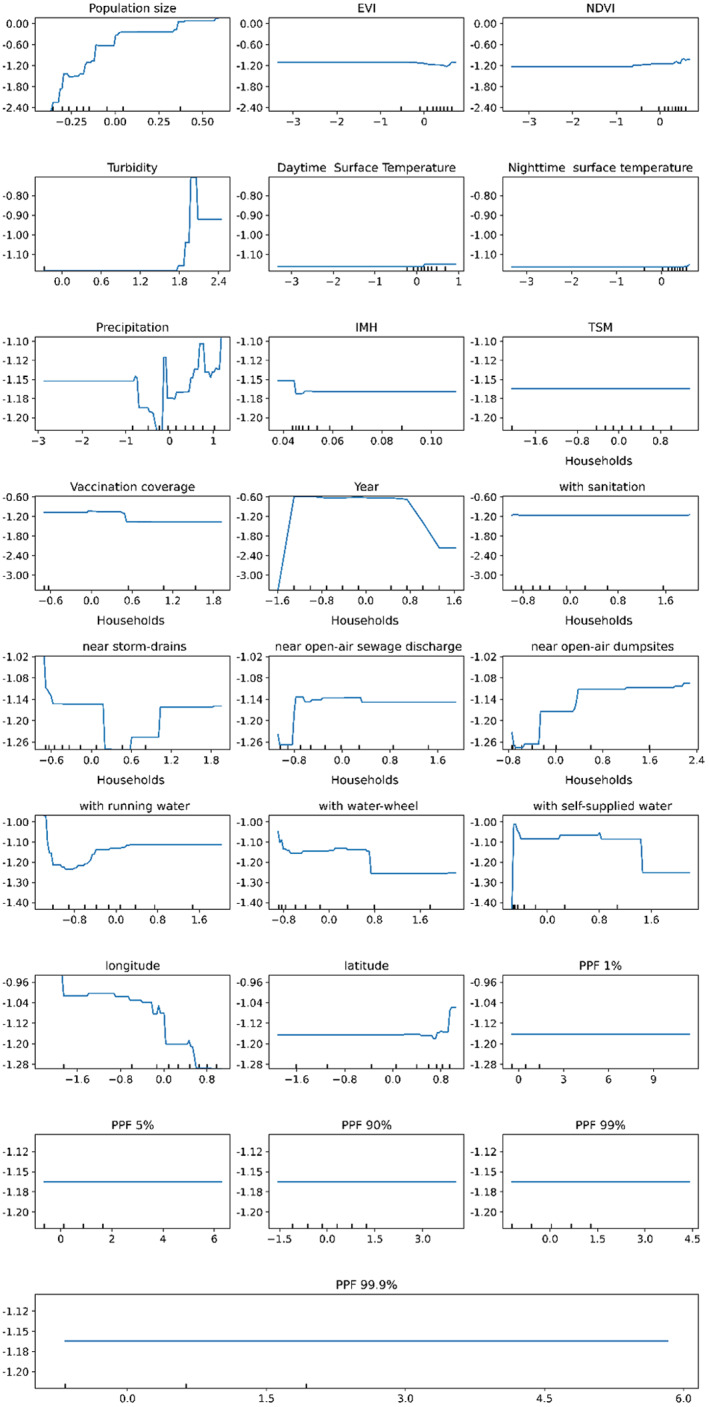
Results of the partial dependence analysis of the explanatory variables for hepatitis‐A from the Histogram Radiant Boost (HGB) regression model. Marks on the *x*‐axis indicate the data distribution.

After selecting the HGB model, a partial dependence analysis (PDA) was applied to indicate the relative dependence of each variable. The results of the PDA reflected how each variable related to hepatitis‐A transmission. PDA values varied between −2.4 and 0 (Figure 2). Positive relations were observed for population size, households near open‐air sewage discharge, households near open‐air dumpsites, and latitude. Negative relations were observed for vaccination coverage, households with public water supply, households with waterwheels, and the municipalities' centroid longitude. A constant relation was observed for the variable households with sanitation. More complex (nonlinear) relations were observed for the variables households near storm‐drains, households with local water supply and year of notification. The dependences of households near storm‐drains, households with local water supply and year of notification presented a bell‐shaped pattern, indicating that they varied depending on the municipality and/or period studied.

The environmental variables with positive relations were turbidity, precipitation and NDVI (the latter one in lesser degree) (Figure 2). IMH and EVI were negatively related. A constant relation was observed for SDT, SNT, TSM, and all PPF derived variables. For normalized turbidity values below 1.8, a partial dependence plot of turbidity indicated no clear relationship with disease transmission, but for higher values partial dependence was positively related to disease transmission. Precipitation and NDVI relative dependences were nonlinearly associated with disease transmission, although they denoted an average positive trend. With respect to precipitation, there was nearly constant partial dependence for below‐average values; for near average values, precipitation had a negative dependence effect for above average values, precipitation had positive dependence. In respect to NDVI, for below zero normalized values, NDVI denoted constant dependence with disease transmission; for higher values, .. dependence was positive. EVI denoted an inverse pattern with respect to NDVI.

**Figure 3:**
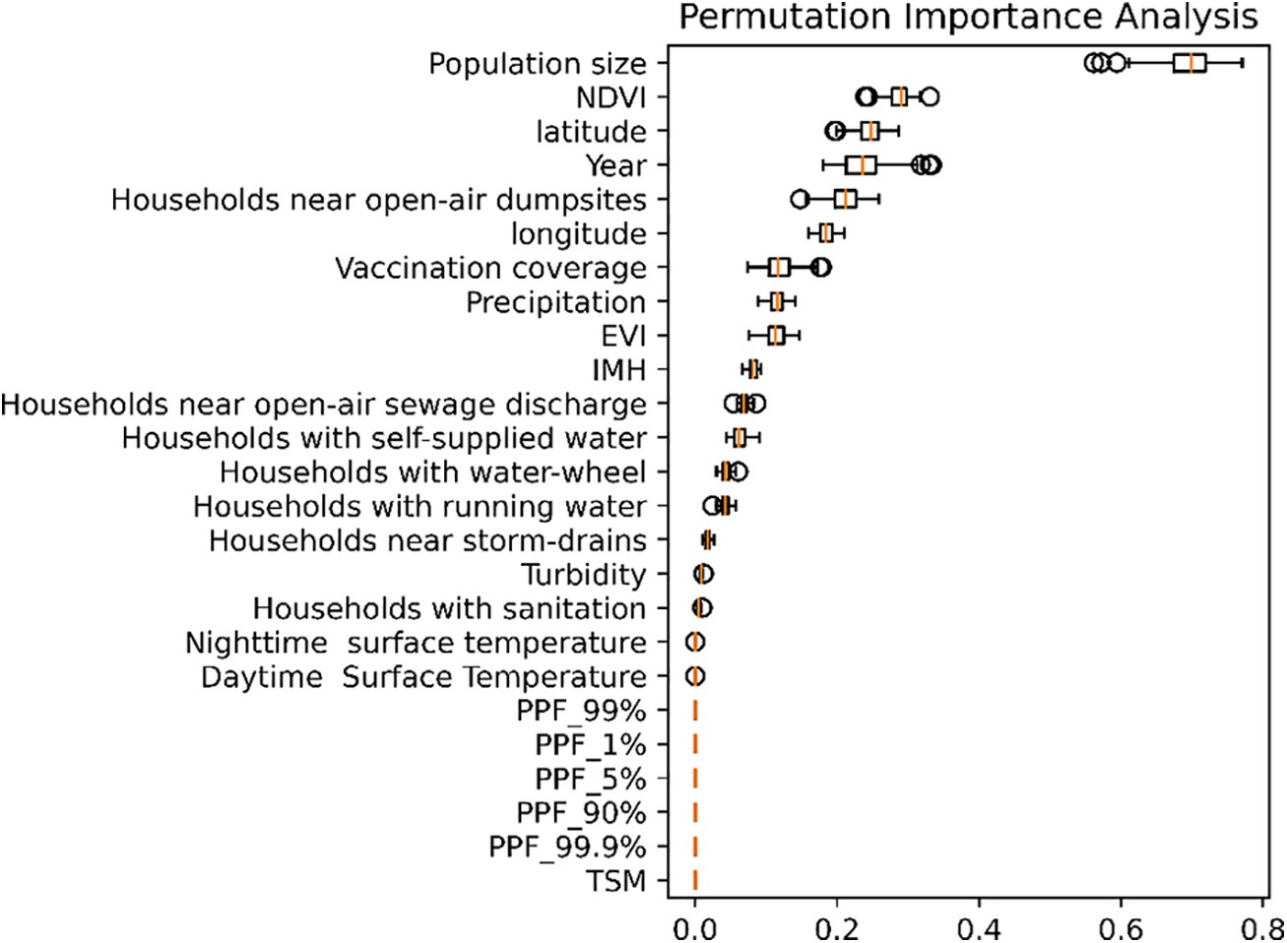
Permutation feature importance analysis depicting the relative importance of each covariate in the Histogram Radiant Boost (HGB) model. Shuffling effort: 99 times.

PFI analysis depicted the relative importance of each environmental and sociodemographic parameter in the HGB model (Figure 3). In decreasing order of importance, population size,NDVI, latitude, year of notification and households near open‐air dumpsites were the five most significant variables in the model. TSM and all PPF variables were the least significant variables in the model. Uncertainty with regard to PFI values were similar; in decreasing order of uncertainty, the variables were population size, year of notification, vaccination coverage and households near open‐air dumpsites.

**Figure 4:**
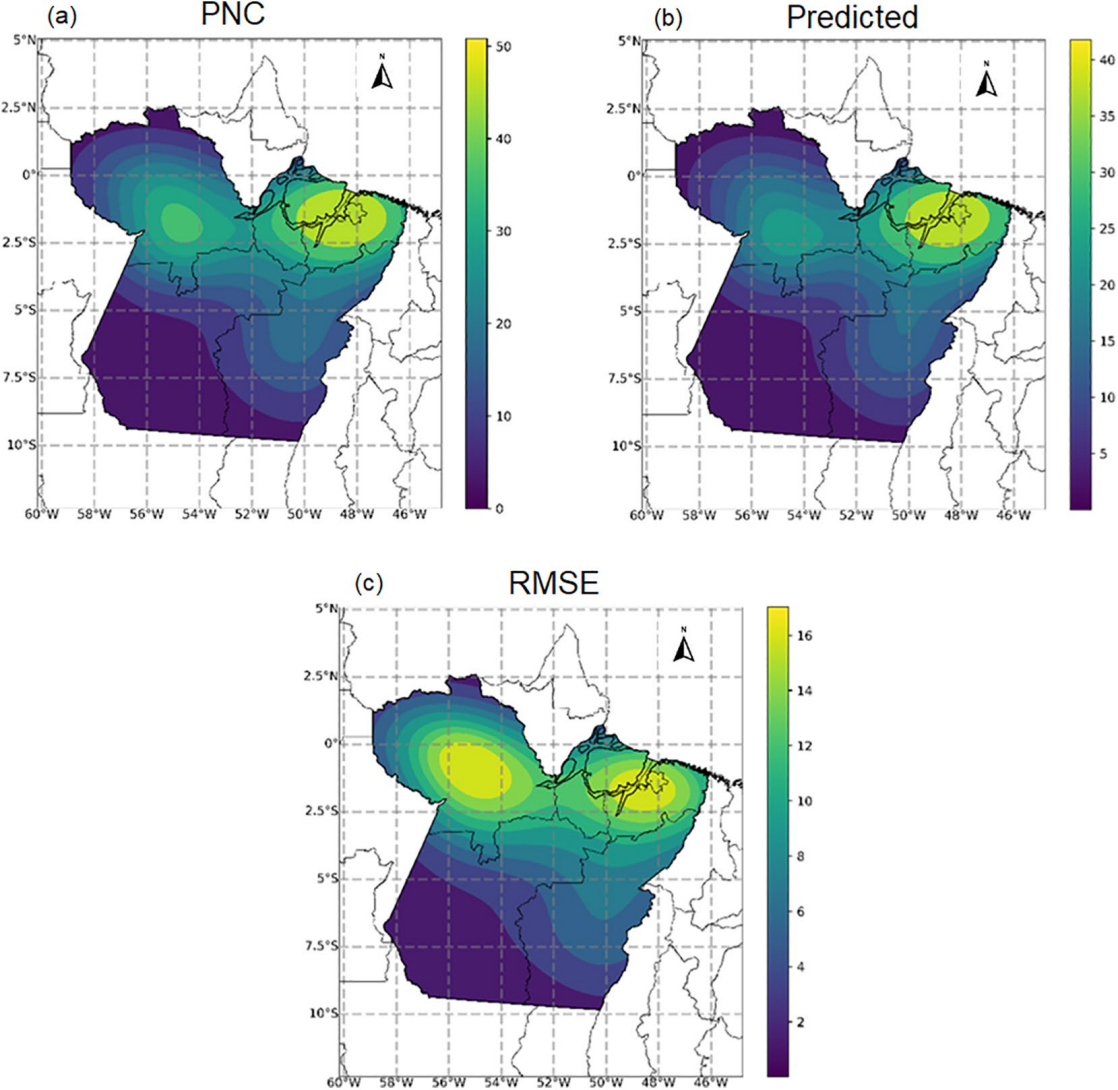
Temporal averages of hepatitis‐A notification cases (upper left panel), Histogram Radiant Boost (HGB) predictions (upper right panel) and HGB residues (lower panel). Choropleths are in quantile format. Choropleths scales are specific to each panel.

The spatial distribution of the notification cases of hepatitis‐A (Figure 4a) indicated two major hotspots, each indicating a high‐risk region for the disease transmission: one northwest and another northeast of the study area. HGB predictions also evidenced these same hotspots (Figure 4b). The residues from the HGB (Figure 4c) were also more densely located at northwest and northeast of the study area, potentially reflecting a spatial structure in the model's residue (Anselin et al., 2006; Ywata & Albuquerque, 2011).

## 4 Discussion

This study evaluated hepatitis‐A transmission by means of sociodemographic and environmental parameters from the state of Pará, Brazil, for the period between January 2008 and December 2017. The observed relations were mostly complex, indicating that multiple interaction effects control the disease transmission. The sociodemographic variables closest related to hepatitis‐A were the population size, national public vaccination coverage, longitude of the municipality centroids, year of notification and location of households near open‐air dumpsites and near storm‐drains. The environmental variables most related to hepatitis‐A were turbidity and precipitation.

Given the importance of public vaccination in mitigating hepatitis‐A transmission (Fiore et al., 2006; WHO, 2011), the vaccination relative dependence values were expected to be higher, if not the highest of all variables of the model. The observed low relative dependence was associated with the insufficient coverage rate of the public vaccination program (MS, 2014), as well as with problems arising from lack of sanitation, sewage disposal and drinking water in the study area (Freitas et al., 2015; IBGE, 2011; UN, 2007). The longitude of the municipality centroids showed that disease transmission is spatially dependent. Westerly municipalities (longitudes < 50°W) had higher risk of hepatitis‐A transmission than easterly municipalities (longitudes > 50°W), a spatial pattern that reflects the sociodemographic characteristics of the study area, where relatively richer and more developed municipalities tend to be located on the eastern part of the state (GOVERNO DO PARÁ, 2010). These findings reinforce the importance of clean drinking water and proper sociodemographic conditions for controlling hepatitis‐A transmission (Jacobsen & Koopman, 2005).

Households near storm‐drains were both negatively and positively related to hepatitis‐A incidence. For municipalities with a low percentage of households near storm‐drains, the relationship was negative, whereas positive dependence was observed for municipalities with high percentage of households near storm‐drains. This pattern was associated with population density, storm‐drain clogging and contact rate of the population with contaminated water‐bodies. A similar dual pattern was observed in a previous study, in which the authors suggested that a variable's dependence duality is a reflection of internal spatial variations of disease transmission in the study area (Rogers, 2000). This reinforces the notion that epidemiological programs, policy‐making and strategy planning must be specific to each area/community (WHO, 2014, 2017). Only then, it is possible to properly consider the unique epidemiological factors associated with a disease's transmission.

Turbidity had a complex relationship with disease transmission and its dependence pattern was expressed by a peaked Gaussian distribution shape. Lower values of turbidity did not influence disease transmission; for average values, the turbidity was positively associated; and for higher values turbidity was negatively related to disease transmission. The peaked Gaussian distribution shape dependence was attributed to different characteristics of the limnological environment, for example, increased untreated sewage discharge into the environment (Guimaraens & Codeço, 2005), contamination of waterbodies nearby, and particle sedimentation (James et al., 2013; UNESCO, 1982). Aside from the fact that untreated sewage is directly linked to virus dispersion and propagation of the disease (Guimaraens & Codeço, 2005), wastewater also influences the attenuation of light in the water column, increasing the turbidity of the water body (de Oliveira et al., 2018). The higher the turbidity, the more suspended particles there are in the water column (Ellison et al., 2014; Jafar‐Sidik et al., 2017; Pereira Filho et al., 2013). Also, more suspended particles in the water column mean a greater adherence rate of other materials (organic and inorganic), leading to an increase in sedimentation rates (Galvez & Niell, 1993; Thornton, 1990; de Wilde, 2013). As a consequence, the suspended particles may act as binding agents in the limnological environment; in sufficiently large number, these particles can more efficiently bind particles like the hepatitis‐A virus (Kendall et al, 2012), increasing its deposition rate. If there are less HAV available in the system, the chances of infection are reduced, directly diminishing disease transmission. In some cases, increases in turbidity can also be related to increases in water turbulence (Knoblauch, 1999). As turbulence increases, higher dispersion forces act on the HAV present in the water column (Simons & Sentürk, 1976). As a consequence, turbulence acts as a cleaning agent that diminishes the virus pool available for potential infection (Gurjão, 2015; Simons & Sentürk, 1976).

Precipitation also denoted a nonlinear association with disease transmission. For below average precipitation, the effect was nearly constant; for near average values, precipitation had a negative effect on disease transmission, while above average precipitation had a positive effect. Lower precipitation events induce less turbulent behavior in water bodies, and consequently a higher deposition rate (Bittencourt‐Oliveira et al., 2012; Pereira Filho et al., 2013). Under this scenario, HAV is expected to be less present in water systems. The opposite is also true. Under higher precipitation events, the deposition rate is reduced with the increase in water turbulence (Bittencourt‐Oliveira et al., 2012; Pereira Filho et al., 2013). Under intense precipitation events, there is contamination of public water supply systems due to increased run‐off from surrounding areas, to inundation processes and/or to flushing of streets, ponds and other potential water sources (Cann et al., 2013). Given that contaminated water serves as a source for the spread of hepatitis‐A (de Paula et al., 2007), therefore, these precipitation events are deemed of great importance for hepatitis‐A transmission (Marcheggiani et al., 2010).

Previous studies have related hepatitis‐A transmission to extreme precipitation and flooding events (Gullón et al., 2017; Marcheggiani et al., 2010). This study, however, by applying the PPF methodology to the study area, found no statistical evidence supporting such a statement. Despite the different tested PPFs, their respective relative dependencies were constant for the disease transmission. Several aspects intrinsic to the study area can be accounted for this poor association. Pará is characterized by an equatorial climate, with daily precipitations (FAPESPA, 2018), with mean annual accumulated precipitation potentially reaching 13.2 m, depending on the subregion (Lima et al., 2010). Pará's have no public management directives regarding its waterbodies, nor even state planning or public billing policy for water usage (ANA, 2013a). Only 25% of all Pará's municipalities have 55% or more of its sewage collected and treated (ANA, 2013b). Furthermore, a great parcel of Pará population have an intrinsic relationship with the local water resources, whether for personal consumption or for public transport (transportation by water) (Menezes et al, 2015). This latter is even more pronounced for riverine communities, whose residents live mostly in Palafita households (Menezes et al, 2015). Residences that are mostly build of wood (when on land) or over floating devices (Gama et al., 2018). Given these intrinsic characteristics of the study area, intense precipitation events can have a positive relationship with the disease transmission, as previously observed for the amazon region (de Paula et al., 2007), and abroad (Gullón et al., 2017; Marcheggiani et al., 2010), but may be masked by these intrinsic characteristics of Pará environment and its local communities.

As disaster events may impact public health in different time frames—from short‐lasting impacts (hours) to long‐lasting ones (years) (Freitas et al., 2015), a time lag effect can impinge a direct assessment of the disease transmission. Thus, future studies are required to investigate this temporal dependence. Other methodological approaches as the Auto Regressive Integrated Moving Average (ARIMA) and artificial neural network models might be possible alternatives (Chadsuthi et al., 2012; Guan et al., 2004; Luz et al., 2008; Ture & Kurt, 2006). Furthermore, given the variability and lack of consensus on how to measure and depict extreme precipitation events (Gullón et al., 2017), other methodological approaches to detect extreme events are required to properly assess a potential relationship with the hepatitis‐A transmission.

Regarding the spatial analyses, the estimated transmission values derived from the HGB model were in agreement with the observed notification cases of hepatitis‐A. The model's results reinforce the notion that the hepatitis‐A transmission is spatially and temporally dependent in the study area. The observed hotspots for the disease transmission followed along with the spatial distribution of the population density (IBGE, 2017), implying that regions of higher density have higher notification cases for the disease. A relationship that is mainly caused by an irregular accessibility of healthcare centers and public vaccination coverage throughout the study area (Affonso et al., 2016; Fernandes & Fernandenos, 2013; Fernandenos & Fernandes 2016). In response to the precarious coverage rate of the national vaccination program in Pará state (Brito & Souto, 2020), one can expect an increase in notification cases for hepatitis‐A in the next years in the study area. Given the spatial complexity inherent to disease transmission modelling in population dynamics (Diez‐Roux, 2000; WHO, 2014), especially regarding waterborne diseases as hepatitis‐A, more studies are required in order to evaluate these spatial structures and its spatial dependency for a more robust disease risk assessments.

The present study reiterates how important it is for public health practitioners and water companies to be aware of the risks related to waterborne disease outbreaks. It is important to stress that the methods applied here can also be extended to other waterborne diseases, reinforcing the applicability of this work. Furthermore, future studies may also apply the current methods for different time‐periods of a same study area (i.e., prior and after national public vaccination programs). Through this temporal segmentation approach, these studies may evidence potential temporal variations in the sociodemographic and environmental factors on the hepatitis‐A transmission. Specifically regarding Brazil, there may be at least three major time‐periods that could be further analyzed: before the public vaccination program (before 2014); between 2014 and 2016, period prior to the Bill N° 204–2016; and after 2016, period in which Bill N° 204–2016 was already operational, potentially resulting in a significant improvement in the compulsory notification system (virtually increasing the hepatitis‐A notification cases).

Given the impacts of extreme weather events on waterborne diseases, especially under a scenario of climate change, health disparities are likely to occur in the near future. A population's ability to adapt to and limit the effects of such events is likely dependent on socioeconomic and environmental circumstances, as well as on the information and technology available (Gullón et al., 2017). Since waterborne diseases are expected to have higher incidence, and even higher geographical coverage due to climate change (Ahern et al., 2005; Davies et al., 2015; UN, 2007), and, given the increase in population density and the lack of proper sanitation and vaccination in developing countries as Brazil (IBGE, 2016, 2018; Paungartten et al., 2015), this essay may be of interest for early warning planning in the public health sector (FORD et al., 2009).

## 5 Conclusions

This study assessed the relationship between hepatitis‐A transmission and environmental and sociodemographic variables in the state of Pará, Brazil. Generalized linear and nonlinear models were examined as alternative predictors for hepatitis‐A. The best‐suited model was the HGB. Population size, lack of sanitation and of proper public vaccination, households' proximity to open‐air dumpsites and storm‐drains, and insufficient access to healthcare facilities and hospitals were the sociodemographic parameters more closely related to HAV transmission. Turbidity and precipitation were the environmental parameters more closely related to disease transmission, and it was found that hepatitis‐A transmission was positively associated with periods of average turbidity and more intense precipitation.

Despite enhancements in the public healthcare sector, Pará state still lacks proper sociodemographic conditions (sanitation, sewage disposal, accessibility to potable water, public education, public awareness, etc.) in order to effectively control the hepatitis‐A without the constant support of the public vaccination programs. A proper mitigation will only be possible if investments are made in alternative strategies for sustained disease control and relief, which are essential for public health policymakers, vaccine developers and disease control specialists to make robust estimates of current and future distribution of disease transmission around the world. Since remote sensing can be of great importance to assess disease‐related environment factors, providing meaningful insights for controlling disease transmission, this study stresses the need to incorporate remote sensing data to epidemiological modelling and surveillance plans in order to develop early prevention strategies for waterborne diseases.

This work emphasizes the importance of incorporating different methodological approaches in epidemiological studies in order to assess the factors mostly related to waterborne diseases transmission. The present study can contribute significantly to preventive strategies aiming the mitigation of the disease transmission in municipalities under higher risk. Here, we reiterate that the applied methods can be extended to other waterborne infectious diseases (i.e., leishmaniosis, harmful algal blooms related infections, diarrhea, and many others). The hepatitis‐A was used as a test case due to its importance to the study area, and due to its standardized database. Future studies can also apply these same methods for different time‐periods in order to assess temporal variations in the regulatory factors of the hepatitis‐A transmission.

## Conflict of Interest

The authors declare no conflicts of interest relevant to this study.

## Data Availability

The data sets here applied can be accessed by means of different sources, as follows: (a) The remote sensing data sets can be directly accessed by means of the Google Earth Engine (GEE) platform. (b) The sociodemographic data set can be directly accessed by means of the Brazilian Institute of Geography and Statistics (IBGE), through the url: https://geoftp.ibge.gov.br/. (c) The epidemiological data set is available by means of the State Health Department of Pará (SESPA). In addition, the above three data sets were merged into a single final data set, which is available at: Leal, Philipe (2021), “Associations Between Environmental and Sociodemographic Data and Hepatitis‐A Transmission in Pará State, (Brazil)”, Mendeley Data, V1, https://doi.org/10.17632/ww35ghv6gx.1. This final (merged) data set is fully pre‐processed, filtered and concatenated, and all respective personal data omitted. The Geoprocessing Laboratory of the Evandro Chagas Institute of the Ministry of Health is authorized by State Health Department of Pará (SESPA) to use and publish data from the Notifiable Diseases Information System (SINAN) and Epidemiological Surveillance Information System (SIVEP).

## References

[gh2229-bib-0002] Affonso, A. G. , Escada, M. I. S. , Amaral, S. , Souza, A. R. , Siqueira, J. M. , Torres, N. M. , et al. (2016). As comunidades ribeirinhas do baixo Tapajós (PA): Infraestrutura, mobilidade, serviços sócio ambientais e conectividade. Instituto Nacional de Pesquisas Espaciais. Retrieved from http://mtc-m21b.sid.inpe.br/col/sid.inpe.br/mtc-m21b/2016/08.02.12.48/doc/publicacao.pdf

[gh2229-bib-0003] Ahern, M. , Kovats, R. S. , Wilkinson, P. , Few, R. , & Matthies, F. (2005). Global health impacts of floods: Epidemiologic evidence. Epidemiologic Reviews, 27(1), 36–46. 10.1093/epirev/mxi004 15958425

[gh2229-bib-0004] Alcântara, E. , Curtarelli, M. , Kampel, M. , & Stech, J. (2016a). Spatiotemporal total suspended matter estimation in Itumbiara reservoir with Landsat‐8/OLI images. International Journal of Cartography, 2(2), 148–165. 10.1080/23729333.2016.1179864

[gh2229-bib-0005] Alcântara, E. , Curtarelli, M. , & Stech, J. (2016b). Estimating total suspended matter using the particle backscattering coefficient: Results from the Itumbiara hydroelectric reservoir (Goiás State, Brazil). Remote Sensing Letters, 7(4), 397–406. 10.1080/2150704x.2015.1137646

[gh2229-bib-0006] ANA . (2013a). Atlas esgotos: Despoluição de bacias hidrográficas Brasil. Retrieved from http://metadados.ana.gov.br/geonetwork/srv/pt/metadata.show?id=471&currTab=distributionAcessoem

[gh2229-bib-0007] ANA . (2013b). Conjuntura dos recursos hídricos no Brasil 2013. Revista de Administração, 48, 432.

[gh2229-bib-0008] Anselin, L. , Syabri, I. , & Kho, Y. (2006). GeoDa: An introduction to spatial data analysis. Geographical Analysis, 38, 5–22. 10.1111/j.0016-7363.2005.00671.x

[gh2229-bib-0009] Avanzi, V. M. , Fonzar, U. J. V. , Silva, E. S. , Teixeira, J. J. V. , & Bertolini, D. A. (2018). Risk areas for hepatitis A, B and C in the municipality of Maringá, Paraná state, Brazil 2007–2010. Geospatial Health, 13(607), 188. 10.4081/gh.2018.607 29772892

[gh2229-bib-0010] Bales, R. C. , Li, S. , Maguire, K. M. , Yahya, M. T. , & Gerba, C. P. (1993). MS‐2 and poliovirus transport in porous media: Hydrophobic effects and chemical perturbations. Water Resources Research, 29(4), 957–963. 10.1029/92wr02986

[gh2229-bib-0011] Barbosa, V. S. , Loyo, R. M. , Guimarães, R. J. P. S. , & Barbosa, C. S. (2017). Os Sistemas de Informação Geográfica em estudo sobre a esquistossomose em Pernambuco. Revista de Saúde Pública, 51, 1–10.28099550

[gh2229-bib-0012] Bittencourt‐Oliveira, M. , Dias, S. , Moura, A. , Cordeiro‐Araújo, M. , & Dantas, E. (2012). Seasonal dynamics of cyanobacteria in a eutrophic reservoir (Arcoverde) in a semi‐arid region of Brazil. Brazilian Journal of Biology, 72(3), 533–544. 10.1590/s1519-69842012000300016 22990824

[gh2229-bib-0013] Boehmke, B. , & Greenwell, B. (2019). Hands‐on machine learning with scikit‐learn, keras and tensor flow. CRC Press.

[gh2229-bib-0014] Brito, W. I. D. E. , & Souto, F. J. D. (2020). Vacinação universal contra hepatite A no Brasil: Análise da cobertura vacinal e da incidência cinco anos após a implantação do programa. Revista Brasileira de Epidemiologia, 23, e200073. 10.1590/1980-549720200073 32638856

[gh2229-bib-0015] Camuffo, D. , della Valle, A. , & Becherini, F. (2018). A critical analysis of the definitions of climate and hydrological extreme events. Quaternary International, 538, 5–13.

[gh2229-bib-0016] Cann, K. F. , Thomas, D. R. , Salmon, R. L. , Wyn‐Jones, A. P. , & Kay, D. (2013). Extreme water‐related weather events and waterborne disease. Epidemiology and Infection, 141(4), 671–686. 10.1017/s0950268812001653 22877498 PMC3594835

[gh2229-bib-0017] Carballo, M , Cody, R. , Kelly, M. , Hatzakis, A. , Thomas, H. C. , Lok, A. S. , et al. (2013). Migration, Hepatitis B, and Hepatitis C. In Viral Hepatitis. 4th Ed., (p. 506–514). Wiley Blackwell

[gh2229-bib-0018] Chadsuthi, S. , Modchang, C. , Lenbury, Y. , Iamsirithaworn, S. , & Triampo, W. (2012). Modeling seasonal leptospirosis transmission and its association with rainfall and temperature in Thailand using time‐series and ARIMAX analyses. Asian Pacific Journal of Tropical Medicine, 5(7), 539–546. 10.1016/s1995-7645(12)60095-9 22647816

[gh2229-bib-0019] Clemens, S. A. C. , Fonseca, J. C. D. , Azevedo, T. , Cavalcanti, A. , Silveira, T. R. , Castilho, M. C. , & Clemens, R. (2000). Soroprevalência para hepatite A e hepatite B em quatro centros no Brasil. Revista da Sociedade Brasileira de Medicina Tropical, 33(1), 01–10. 10.1590/s0037-86822000000100001 10881112

[gh2229-bib-0020] Curriero, F. C. , Patz, J. A. , Rose, J. B. , & Lele, S. (2001). The association between extreme precipitation and waterborne disease outbreaks in the United States, 1948‐1994. American Journal of Public Health, 91(8), 1194–1199. 10.2105/ajph.91.8.1194 11499103 PMC1446745

[gh2229-bib-0021] da Silva, V. S. , Salami, G. , da Silva, M. I. O. , Silva, E. A. , Monteiro Junior, J. J. , & Alba, E. (2019). Methodological evaluation of vegetation indexes in land use and land cover (LULC) classification. Geology, Ecology, and Landscapes, 0, 1–11.

[gh2229-bib-0022] Davies, G. I. , McIver, L. , Kim, Y. , Hashizume, M. , Iddings, S. , & Chan, V. (2015). Water‐borne diseases and extreme weather events in Cambodia: Review of impacts and implications of climate change. International Journal of Environmental Research and Public Health, 12(1), 191–213. 10.3390/ijerph120100191 PMC4306857 25546280

[gh2229-bib-0023] de Paula, V. S. , Diniz‐Mendes, L. , Villar, L. M. , Luz, S. L. B. , Silva, L. A. , Jesus, M. S. , et al. (2007). Hepatitis A virus in environmental water samples from the Amazon Basin. Water Research, 41(6), 1169–1176. 10.1016/j.watres.2006.11.029 17306323

[gh2229-bib-0024] Diaz, H. F. , & Murnane, R. J. (2008). Preface: The significance of weather and climate extremes to society: An introduction. Climate Extremes and Society, xiii–xvi. 10.1017/CBO9780511535840.002

[gh2229-bib-0025] Diez‐Roux, A. V. (2000). Multilevel analysis in public health research. Annual Review of Public Health, 21, 171–192. 10.1146/annurev.publhealth.21.1.171 10884951

[gh2229-bib-0026] Dogliotti, A. I. , Ruddick, K. G. , Nechad, B. , Doxaran, D. & Knaeps, E. (2015). A single algorithm to retrieve turbidity from remotely‐sensed data in all coastal and estuarine waters. Remote Sensing of Environment, 156, 157–168. 10.1016/j.rse.2014.09.020

[gh2229-bib-0027] Ellison, C. A. , Savage, B. E. , & Johnson, G. D. (2014). Suspended‐sediment concentrations, loads, total suspended solids, turbidity, and particle‐size fractions for selected rivers in Minnesota, 2007 through 2011: U. S Geological Survey Scientific Scientific Investigations Report 2013–5205. 10.3133/sir20135205

[gh2229-bib-0028] FAPESPA . (2018). Anuário Estatístico do Pará. (p. 1.) FAPESPA

[gh2229-bib-0086] Farr, T. G. , Rosen, P. A. , Caro, E. , Crippen, R. , Duren, R. , Hensley, S. , et al. (2007). The shuttle radar topography mission. Reviews of Geophysics, 45(2). 10.1029/2005rg000183

[gh2229-bib-0029] Federal Aviation Administration (FAA) . (2003). Using modern computing tools to fit the Pearson type III distribution to aviation loads data. Retrieved from https://Dot/Faa/Ar-03/62

[gh2229-bib-0030] Fernandes, A. S. , Fernandesnos, A. P. A. (2013). A acessibilidade nos transportes: A realidade das comunidades ribeirinhas da amazônia paraense. In Anais do Congresso Brasileiro de Educação Especial. Anais. Retrieved from https://proceedings.science/cbee/cbee6/papers/a-acessibilidade-nos-transportes--a-realidade-das-comunidades-ribeirinhas-da-amazonia-paraense#download-paper

[gh2229-bib-0031] Fernandesdos, A. P. C. S. , & Fernandes, A. S. A. (2016). Acessibilidade nos transportes e as pessoas com deficiência da comunidade ribeirinha da amazônia paraense. Revista Cocar, 10(19), 240–264.

[gh2229-bib-0032] Fiore, A. E. , Wasley, A. , & Bell, B. P. (2006). Prevention of hepatitis A through active or passive immunization: Recommendations of the Advisory Committee on Immunization Practices (ACIP). Coordinating Center for Health Information and Service, Centers for Disease Control and Prevention (CDC), U.S. Department of Health and Human Services. Retrieved from http://www.ncbi.nlm.nih.gov/pubmed/16708058 16708058

[gh2229-bib-0033] Fonseca, F. R. , Saraiva, T. S. , Freitas, C. C. , Dutra, L. V. , Monteiro, A. M. V. , Rennó, C. D. , et al. (2007). Desenvolvimento de um índice hidrológico para aplicação em estudos de distribuição da prevalência de esquistossomose em Minas Gerias. Anais XIII Simpósio Brasileiro de Sensoriamento Remoto, 2589–2595.

[gh2229-bib-0034] Ford, T. E. , Colwell, R. R. , Rose, J. B. , Morse, S. S. , Rogers, D. J. , & Yates, T. L. (2009). Using satellite images of environmental changes to predict infectious disease outbreaks. Emerging Infectious Diseases, 15(9), 1341–1346. 10.3201/eid1509.081334 19788799 PMC2819876

[gh2229-bib-0035] Fox, J. (2008). Applied regression and generalized linear models. In Applied regression analysis and generalized linear models (2nd ed., pp. 379–424). Sage Publications, Inc.

[gh2229-bib-0036] Freitas, C. M. D. , Silva, D. R. X. , Sena, A. R. M. D. , Silva, E. L. , Sales, L. B. F. , Carvalho, M. L. D. , et al. (2015). Desastres naturais e saúde: Uma análise da situação do Brasil. Ciência & Saúde Coletiva, 19(9), 3645–3656. 10.1590/1413-81232014199.00732014 25184572

[gh2229-bib-0038] Funk, C. , Peterson, P. , Landsfeld, M. , Pedreros, D. , Verdin, J. , Shukla, S. , et al. (2015). The climate hazards infrared precipitation with stations ‐ A new environmental record for monitoring extremes. Scientific Data, 2(1), 1–21. 10.1038/sdata.2015.66 PMC4672685 26646728

[gh2229-bib-0037] Funk, C. C. , Peterson, P. J. , Landsfeld, M. F. , Pedreros, D. H. , Verdin, J. P. , Rowland, J. D. , et al. (2014). A quasi‐global precipitation time series for drought monitoring. U.S. Geological Survey Data Series, 832(4), 1–12.

[gh2229-bib-0039] Galvez, J. A. , & Niell, F. X. (1993). Sedimentation and Mineralization of Seston in a eutrophic reservoir, with a tentative sedimentation model. In M. Straskraba , J. G. Tundisi , & A. Duncan , (Eds.), Developments in Hydrology: Comparative Reservoir Limnology and Water Quality Management (pp. 119–126). Kluwer Academic Publishers. 10.1007/978-94-017-1096-1_7

[gh2229-bib-0040] Gama, A. S. M. , Fernandes, T. G. , Parente, R. C. P. , & Secoli, S. R. , (2018). Inquérito de saúde em comunidades ribeirinhas do Amazonas, Brasil. Cadernos de Saúde Pública, 34(2), 1–16. 10.1590/0102-311X00002817 29489939

[gh2229-bib-0041] Gorelick, N. Hancher, M. , Dixon, M. , Ilyushchenko, S. , Thau, D. , & Moore, R. (2017) Google Earth engine: Planetary‐scale geospatial analysis for everyone. Remote Sensing of Environment, 202, 18–27.

[gh2229-bib-0042] GOVERNO DO PARÁ . (2010). Síntese do índice de desenvolvimento humano municipal – IDHM para o estado do Pará.

[gh2229-bib-0043] Guan, P. , Huang, D.‐S. , & Zhou, B.‐S. (2004). Forecasting model for the incidence of hepatitis A based on artificial neural network. World Journal of Gastroenterology, 10(24), 3579–3582. 15534910 10.3748/wjg.v10.i24.3579 PMC4611996

[gh2229-bib-0044] Guimaraens, M. A. D. , & Codeço, C. T. (2005). Experiments with mathematical models to simulate hepatitis A population dynamics under different levels of endemicity. Cadernos de Saúde Pública, 21(5), 1531–1539. 10.1590/s0102-311x2005000500026 16158159

[gh2229-bib-0045] Gullón, P. , Varela, C. , Martínez, E. V. , & Gómez‐Barroso, D. (2017). Association between meteorological factors and hepatitis A in Spain 2010–2014. Environment International, 102, 230–235. 10.1016/j.envint.2017.03.008 28325534

[gh2229-bib-0046] Gurjão, T. C. M. (2015). GENÓTIPOS DO VÍRUS DA HEPATITE A (VHA) DETECTADOS EM DIFERENTES ECOSSISTEMAS AQUÁTICOS E A RELAÇÃO DO VHA COM OS INDICADORES DE QUALIDADE DA ÁGUA, BELÉM, PARÁ, BRASIL. Universidade Federal do Pará Dissertação (Mestrado em Biologia)—Brasil.

[gh2229-bib-0047] Hu, Z. , Xiao, Q. , Yang, J. , Xiao, W. , Wang, W. , Liu, S. , & Lee, X. (2015). Temporal dynamics and drivers of ecosystem metabolism in a large subtropical Shallow Lake (Lake Taihu). International Journal of Environmental Research and Public Health, 12(4), 3691–3706. 10.3390/ijerph120403691 25837347 PMC4410210

[gh2229-bib-0048] IBGE . (2011). Censo demográfico 2010: Características da população e dos domicílios: resultados do universo.

[gh2229-bib-0049] IBGE . (2016). Arranjos Populacionais e Concentrações Urbanas do Brasil.

[gh2229-bib-0050] IBGE . (2017). Estimativas da população residente no Brasil e Unidades da Federação em 1° de julho de 2017. Retrieved from ftp://ftp.ibge.gov.br/Estimativas_de_Populacao/Estimativas_2017/estimativa_dou_2017.pdf

[gh2229-bib-0051] IBGE . (2018). Estimativas de População. Retrieved from https://www.ibge.gov.br/estatisticas-novoportal/sociais/populacao/9103-estimativas-de-populacao.html?=&t=downloads

[gh2229-bib-0052] IBGE . (2019). Divisões politico‐administrativas do Brasil.

[gh2229-bib-0053] Jacobsen, K. H. , & Koopman, J. S. (2005). The effects of socioeconomic development on worldwide hepatitis A virus seroprevalence patterns. International Journal of Epidemiology, 34(3), 600–609. 15831565 10.1093/ije/dyi062

[gh2229-bib-0054] Jafar‐Sidik, M. , Gohin, F. , Bowers, D. , Howarth, J. , & Hull, T. (2017). The relationship between suspended particulate matter and turbidity at a mooring station in a coastal environment: Consequences for satellite‐derived products. Oceanologia, 59(3), 365–378.

[gh2229-bib-0055] James, L. A. & Lecce, S. A. (2013). Impacts of land‐use and land‐cover change on river systems. In J. F. Shroder, (Ed.). Treatise on Geomorphology. (pp. 768–793). Academic Press.

[gh2229-bib-0056] Justice, C. O. , Vermote, E. , Townshend, J. R. , Defries, R. , Roy, D. P. , Hall, D. K. , et al. (1998). The moderate resolution imaging spectroradiometer (MODIS): Land remote sensing for global change research. IEEE Transactions on Geoscience and Remote Sensing, 36(4), 1228–1249.

[gh2229-bib-0057] Kendall, K. , Kendall, M. , & Rehfeldt, F. (2012). Adhesion of cells , viruses and nanoparticles. SPRINGER.

[gh2229-bib-0058] Kingma, D. P. , & Ba, J. L. (2015). Adam: A method for stochastic optimization. In 3rd International conference on learning representations. (pp. 1–15). ICLR 2015 ‐ Conference Track Proceedings.

[gh2229-bib-0059] Knoblauch, H. (1999). Overview of density flows and turbidity currents. Water Resources Research Laboratory.

[gh2229-bib-0060] Lee, Z. P. , Shang, S. , Hu, C. , Du, K. , Weidemann, A. , Hou, W. , et al. (2015). Secchi disk depth: A new theory and mechanistic model for underwater visibility. Remote Sensing of Environment, 169, 139–149.

[gh2229-bib-0061] Lima, A. M. M. , Cruz, F. M. , Cavalcante, L. M. , de Leão, L. M. , Chaves, M. I. J. , & Santos, V. J. C. , et al. (2010). A gestão da oferta hídrica no estado do pará e seus aspectos condicionantes. Revista Brasileira de Recursos Hídricos, 15(3), 69–83.

[gh2229-bib-0062] Luz, P. M. , Mendes, B. V. , Codeço, C. T. , Struchiner, C. J. , & Galvani, A. P. , (2008). Time series analysis of dengue incidence in Rio de Janeiro, Brazil. The American Journal of Tropical Medicine and Hygiene, 79(6), 933–939. 19052308

[gh2229-bib-0063] Marcheggiani, S. , Puccinelli, C. , Ciadamidaro, S. , Della Bella, V. , Carere, M. , Francesca Blasi, M. , et al. (2010). Risks of water‐borne disease outbreaks after extreme events. Toxicological and Environmental Chemistry, 92(3), 593–599.

[gh2229-bib-0064] Mavignier, A. L. , & Frischkorn, H. (1992). *Physical, chemical and bacteriological study of Cocó River, Fortaleza ‐ Ceará. Anais do 1 simpósio de Recursos hídricos do nordeste, Recife, 25‐27 nov*. Anais…Fortaleza.

[gh2229-bib-0065] Menezes, T. M. , Perdigão, A. K. D. A. V. , & Pratschke, A. (2015). O tipo palafita amazônico: Contribuições ao processo de projeto de arquitetura. Oculum Ensaios, 12(2), 237.

[gh2229-bib-0066] Molnar, C. (2019). Interpretable machine learning: A guide for making black box models explainable. Lulu. Retrieved from https://christophm.github.io/interpretable-ml-book/

[gh2229-bib-0067] MS . (2002). *Programa nacional de hepatites virais: avaliação da assistência as hepatites virais no Brasil 2002. 1° edição ed. Brasília ‐ DF*. MINISTÉRIO DA SAÚDE.

[gh2229-bib-0068] MS . (2005). Programa nacional para a prevenção e o controle das hepatites virais: Manual de aconselhamento em hepatites virais. MINISTÉRIO DA SAÚDE. SECRETARIA DE VIGILÂNCIA EM SAÚDE. DEPARTAMENTO DE VIGILÂNCIA EPIDEMIOLÓGICA v. Série D.

[gh2229-bib-0069] MS . (2007). Sistema de informação de agravos de notificação (SINAN): Normas e rotinas (2nd ed.). Brasília: Ministério da Saúde.

[gh2229-bib-0001] MS . (2014). Informe técnico da introdução da vacina adsorvida Hepatite‐A (inativada)Brasília. Brasil: MINISTÉRIO DA SAÚDE. Retrieved from http://portalarquivos2.saude.gov.br/images/pdf/2015/junho/26/Informe-t--cnico-vacina-hepatite-A-junho-2014.pdf

[gh2229-bib-0070] MS . (2018). HEPATITES virais 2018. In Secretaria de Vigilância em Saúde − Ministério da Saúde, Boletim Epidemiológico, [s.l.], MINISTÉRIO DA SAÚDE; SECRETARIA DE VIGILÂNCIA EM SAÚDE. Retrieved from http://portalarquivos2.saude.gov.br/images/pdf/2018/julho/05/Boletim-Hepatites-2018.pdf Acesso em 7 September 2018.

[gh2229-bib-0071] MS . (2019). Base de Dados ‐ DATASUS.

[gh2229-bib-0072] Nunes, H. M. , Soares, M. D. C. P. , Sarmento, V. P. , Malheiros, A. P. , Borges, A. M. , Silva, I. S. D. , & Paixão, J. F. D. (2016). Soroprevalência da infecção pelos vírus das hepatites A, B, C, D e E em município da região oeste do Estado do Pará, Brasil. Revista Pan‐Amazônica de Saúde, 7(1),55–62

[gh2229-bib-0073] Ody, A. , Doxaran, D. , Vanhellemont, Q. , Nechad, B. , Novoa, S. , Many, G. , et al. (2016). Potential of high spatial and temporal ocean color satellite data to study the dynamics of suspended particles in a micro‐tidal river plume. Remote Sensing, 8(3), 245.

[gh2229-bib-0074] Oliveira, A. R. M. de. , Borges, A. C. , Matos, A. T. , & Nascimento, M. , (2018). Estimation on the concentration of suspended solids from turbidity in the water of two sub‐basins in the Doce River basin. Engenharia Agrícola, 38(5), 751–759.

[gh2229-bib-0075] Parsons, K. (2003). Human Thermal Enviroments (2nd Ed.). Taylor & Francis.

[gh2229-bib-0076] Patel, K. (2020). Of mosquitoes and models: Tracking disease by satellite. Retrieved from https://earthobservatory.nasa.gov/features/disease-vector?src=eoa-features

[gh2229-bib-0077] Paungartten, S. P. L. , Bordalo, C. A. L. , & de Lima, A. M. M. (2015). Condições socioeconômicas de bacias hidrográficas: Um estudo de caso na região metropolitana de Belém ‐ pa. Revista GeoAmazônia, 03(06), 83–95.

[gh2229-bib-0078] Pavlov, Y. L. (2019). Random forests (pp. 1–122). Random Forests.

[gh2229-bib-0079] Pedregosa, F. , Varoquaux, G. , Gramfort, A. , Michel, V. , Thirion, B. , Grisel, O. , et al. (2011). Scikit‐learn: Machine learning in python. Journal of Machine Learning Research, 12, 2825–2830.

[gh2229-bib-0081] Pereira, F. E. L. , & Gonçalves, C. S. A. (2003). Hepatite A. Revista da Sociedade Brasileira de Medicina Tropical, 36(3), 387–400. 12908041 10.1590/s0037-86822003000300012

[gh2229-bib-0080] Pereira Filho, W. Santos, F. C. , Cassol, A. P. V. , Domingues, A. L. , & Prado, D. A. (2013). Influência de componentes oticamente ativos relacionados a reservatórios em cascata ‐ Rio Jacuí ‐ RS. In.Anais XVI Simpósio Brasileiro de Sensoriamento Remoto (pp. 9036–9042). INPE

[gh2229-bib-0082] Petrere, M., Jr. , & Friedman, J. (2000). Greedy function approximation: A gradient boosting machine. Annals of Statistics, 29(5), 1189–1232.

[gh2229-bib-0083] Ramalho, H. D. (2020). A caracterização do município como entidade federativa. (pp. 1–20).

[gh2229-bib-0084] Rodrigues, T. , Mishra, D. R. , Alcântara, E. , Watanabe, F. , Rotta, L. , & Imai, N. N. (2017). Retrieving total suspended matter in tropical reservoirs Within a cascade system with widely differing optical properties. IEEE Journal of Selected Topics in Applied Earth Observations and Remote Sensing, 10(12), 5495–5512.

[gh2229-bib-0085] Rogers, D. J. (2000). Satellites, space, time and the african trypanosomiases. Advances in Parasitology, 47, 129–171. 10997206 10.1016/s0065-308x(00)47008-9

[gh2229-bib-0087] Santos, K. D. S. , Guimarães, R. J. D. P. S. , Sarmento, P. S. D. M. , & Morales, G. P. (2019). Perfil da hepatite A no município de Belém, Pará, Brasil. REvista visa em debate, 7(2), 18–27.

[gh2229-bib-0088] Sattar, S. A. , Tetro, J. , Bidawid, S. , & Farber, J. (2000). Foodborne spread of hepatitis A: Recent studies on virus survival, transfer and inactivation. Canadian Journal of Infectious Diseases, 11(3), 159–163. 10.1155/2000/805156 PMC2094762 18159284

[gh2229-bib-0089] Simons, D. B. , & Sentürk, F. (1976). Sediment transport technology. (p. 1). Water Resources Publications.

[gh2229-bib-0090] Smith, K. R. , et al. (2015). Human health: Impacts, adaptation, and co‐benefits. In Climate Change 2014 Impacts, Adaptation and Vulnerability: Part A: Global and Sectoral Aspects. (pp. 709–754). Cambridge University Press.

[gh2229-bib-0091] Souto, F. J. D. , de Brito, W. I. , & Fontes, C. J. F. (2019). Impact of the single‐dose universal mass vaccination strategy against hepatitis A in Brazil. Vaccine, 37(6), 771–775. 30639462 10.1016/j.vaccine.2018.12.054

[gh2229-bib-0092] SRTM . (2015). The shuttle radar topography mission (SRTM) collection user guide. (pp. 1–17)

[gh2229-bib-0093] Thornton, K. W. (1990). Sedimentary processes. In Reservoir limnology: Ecological perspectives. (pp. 43–69). John Wiley & Sons.

[gh2229-bib-0094] Ture, M. , & Kurt, I. (2006). Comparison of four different time series methods to forecast hepatitis A virus infection. Expert Systems with Applications, 31(1), 41–46.

[gh2229-bib-0095] UN . (2007). Climate change: Impacts, vulnerabilities and adaptation in developing countries. United Nations Framework Convention on Climate Change. Retrieved from http://unfccc.int/resource/docs/publications/impacts.pdf

[gh2229-bib-0096] UNESCO . (1982). Sedimentation problems in river basins. In Studies and reports in hydrology (pp. 152).

[gh2229-bib-0097] Unpingco, J. (2016). Python for probability, statistics, and machine learning. Springer.

[gh2229-bib-0098] U.S. GEOLOGICAL SURVEY . (2019). Landsat 8 Surface Reflectance Code (LASRC) Poduct Guide. (No. LSDS‐1368 Version 2.0). (p. 40) May.

[gh2229-bib-0099] Virtanen, P. , Gommers, R. , Oliphant, T. E. , Haberland, M. , Reddy, T. , Cournapeau, D. , et al. (2020). SciPy 1.0: Fundamental Algorithms for Scientific Computing in Python. Nature Methods, 17(3), 261–272. 32015543 10.1038/s41592-019-0686-2 PMC7056644

[gh2229-bib-0100] Vogel, R. W. , & McMartin, D. E. (1991). Probability plot goodness‐of‐fit and skewness estimation procedures for the Pearson type 3 distribution. Water Resources Research, 27(12), 3149–3158.

[gh2229-bib-0101] Wan, Z. , Hook, S. , & Hulley, G. (2015). MOD11A2 MODIS/Terra Land Surface Temperature/Emissivity 8‐Day L3 Global 1km SIN Grid V006.

[gh2229-bib-0102] Waskom, M. (2020). THE SEABORN DEVELOPMENT TEAM. Seaborn Python's Package. Retrieved from 10.5281/zenodo.592845

[gh2229-bib-0103] Wasserman, L. (2009). All of Statistics: A Concise Course in Statistical Inference. (p. 46). Springer Berlin Heidelberg.

[gh2229-bib-0104] WHO . (2009). Protecting health from climate change: Connecting science, policy and people. World Health Organization. Retrieved from https://apps.who.int/iris/bitstream/handle/10665/44246/9789241598880_eng.pdf;jsessionid=76ECF990F9BB0FB66A05CEF32C24613C?sequence=1

[gh2229-bib-0105] WHO . (2011). Evidence based recommendations for use of hepatitis A vaccines in immunization services: Background paper for SAGE discussions. World Health Organization.

[gh2229-bib-0106] WHO . (2014). Gender, climate change and health. WHO Press. Retrieved from https://apps.who.int/iris/bitstream/handle/10665/144781/9789241508186_eng.pdf;jsessionid=FD60C4C0643A7E9306E66D67944C458B?sequence=1

[gh2229-bib-0107] WHO . (2016). WHO: Viral hepatitis 2016–2021. World Health Organization.

[gh2229-bib-0108] WHO . (2017). Global hepatitis report. World Health Organization.

[gh2229-bib-0109] WHO . (2019). Hepatitis A. Retrieved from https://www.who.int/immunization/diseases/hepatitisA/en/

[gh2229-bib-0110] Wilde, P. de. (2013). Neural Network Models: theory and project. (p. 369). Springer.

[gh2229-bib-0111] Ywata, A. X. D. C. , & Albuquerque, P. H. D. M. (2011). Métodos e modelos em econometria espacial uma revisão. Revista Brasileira de Biometria, 29(2), 273–306.

